# Impact of Androstenone on Leash Pulling and Jumping Up in Dogs

**DOI:** 10.3390/ani6050034

**Published:** 2016-05-09

**Authors:** Glenna Pirner, John McGlone

**Affiliations:** Department of Animal and Food Sciences, Texas Tech University, Lubbock, TX 79409, USA; glenna.pirner@ttu.edu

**Keywords:** behavior, canine, interomone, jumping up, leash pulling, pheromone

## Abstract

**Simple Summary:**

Behavior problems such as leash pulling and jumping up are common reasons given for relinquishing dogs to animal shelters. Interomones are chemical signals produced by one species that elicit an effect on a different species; this study examines the effects of androstenone (produced by boars) on dog leash pulling and jumping up behavior. Androstenone in spray form reduces leash pulling behavior compared to a control spray. This interomone may provide instant relief for behavioral problems and it may be used as a natural tool in conjunction with a training program to reduce unwanted behavior such as leash pulling.

**Abstract:**

Dogs are relinquished to shelters due to behavioral problems, such as leash pulling and jumping up. Interomones are chemical cues produced by one species that elicit a response in a different species. We reported earlier that androstenone, a swine sex pheromone, acts as an interomone to reduce barking in dogs. Here we report two models using 10 dogs/study: a dog jumping and a dog walking model. For the leash-pulling model, each time the dog pulled on the leash the walker either did nothing (NOT), or sprayed the dog with water (H_2_O), androstenone + water (ANH), androstenone 0.1 µg/mL (AND1), or androstenone 1.0 µg/mL (AND2). The number of pulls during each walk was counted. For the jumping up model, each time the dog jumped the researcher did nothing (NOT), or sprayed the dog with H_2_O, ANH, AND1, or AND2. The number of jumps and the time between jumps were recorded. In Study 1, ANH, AND1, and AND2 each reduced leash pulling more than NOT and H_2_O (*p* < 0.01). In Study 2, all treatments were effective in reducing jumping up behavior. Androstenone reduced jumping up, but not beyond that elicited by a spray of water alone. We conclude that androstenone in multiple delivery vehicles reduced leash pulling. The burst of air intended as a disruptive stimulus in the correction sprays may be too harsh for more sensitive dogs, and as such use of these sprays is cautioned in these animals. For other dogs, this interomone can be used to stop some behavior immediately or as a part of a training program to reduce undesirable behavior.

## 1. Introduction

Behavioral issues are given as one of the primary reasons dogs are relinquished to shelters in the U.S. [1,2,3]. The most common behavioral issues are aggression, inappropriate elimination, excessive vocalization, and excitability [4]. Excitable behavior such as jumping up or leash pulling by the dog can be not only a nuisance but can potentially be dangerous for both the dog and the owner, especially if the dog is large [5]. In a survey conducted by Blackwell *et al.* [6], jumping up was reported as an undesirable behavior by 78% of owners, and pulling on the leash was reported by 69% of owners. Some dogs may pull against pressure such as that exerted by a collar. The resulting pressure on the neck can damage the larynx or trachea, and also significantly increases intraocular pressures [7,8]. Therefore, leash pulling may be not only annoying, but harmful to both humans and dogs. Unpleasant experiences during walks may result in the owner’s unwillingness to take the pet for walks, leading to more behavior problems due to boredom or unexpended energy. Dogs often jump up as a greeting, to play, and/or to get attention [9]. This behavior can result in the human being scratched or knocked down, especially if the human is young or elderly. Although this is an undesirable behavior, humans recognize the jumping up as a friendly behavior and often unintentionally reinforce it by returning the greeting. Owners using punishments such as yelling or physical correction as training methods reported higher levels of aggression and avoidance in their dogs compared to owners that used non-punishment methods [6]. It is possible for the dog to incorrectly associate the punishment with the owner rather than with the undesirable behavior. As such, development of a training tool that is disruptive rather than punishing may be more beneficial to overall training. A disruptive stimulus differs from positive punishment in that a disruptive stimulus is an undesirable event that prevents or alters the behavior of animals and allows for a transition to a more desirable behavior [10,11]. On the other hand, positive punishment is associated with a stimulus that elicits pain or fear in the animal with the intention of that pain or fear being associated with the particular behavior [12]. 

Pheromones are chemicals that animals use to communicate within a species. Karlson and Luscher [13] described pheromones as “substances secreted to the outside by an individual and received by a second individual of the same species in which they release a specific reaction”. Recent studies have shown that some chemical cues may be detected between species [14]. Clearly, volatile molecules and many receptors for these molecules are conserved across mammalian species. McGlone coined the term “interomone” to describe those molecules that are a pheromone in one species, but may elicit a different, often unpredictable effect in another species [15]. Because pheromones across many species are composed of relatively similar volatile compounds, the interomone may have either a related or completely different effect on the receiving species compared to the sending species. 

Androstenone is a sex pheromone secreted in the saliva of boars to promote acceptance of mounting behavior by the female and it also functions to reduce aggression in group-housed swine [16]. Nobody has yet argued that androstenone is a pheromone in the dog. However, previous work in this laboratory suggests that androstenone does, in fact, have an effect on dog behavior. In spray form, androstenone stopped dogs from barking, suggesting its potential to reduce occurrences of excitable behavior such as jumping up and leash pulling [17]. The objectives of this study were (1) to determine if androstenone spray reduced leash pulling in dogs using a model of dog walking; and (2) to determine if androstenone spray reduced jumping up behavior in dogs using a model of dog jumping.

## 2. Experimental Section

### 2.1. General

All research was conducted after approval of the Texas Tech University Institutional Animal Care and Use Committee (14009-01). Space, management, and care of dogs were consistent with the U.S. Animal Welfare Act. Research was funded by Perrigo Animal Health (24G476). Research was conducted at a municipal adoption shelter in Lubbock, Texas. 

Dogs used in both studies were relinquished to the shelter and deemed to be eligible for adoption based on veterinary examinations and behavior tests conducted by shelter staff. Overly aggressive dogs or dogs with known or suspected health conditions were not candidates. Because dog availability varied with adoptions and new intakes, exact ages and body weights were unknown, but subjects ranged from juveniles (<1 year) to middle-aged adults (>1 year) and weighed at least 9.1 kg. Intact and altered males and females of various breeds and weights were used (Table 1). Each dog experienced only one treatment, and dogs used in the walking study were not used in the jumping up study to eliminate any effect of previous experience with being sprayed. Dogs were housed in 2.4 m × 3.7 m kennels with concrete walls and floors and barred metal doors. Food was provided twice daily and water was available *ad libitum*. Animal daily care was provided by the municipal staff. 

### 2.2. Experimental Design and Treatments

#### 2.2.1. Study 1: Dog Walking Model

**Figure 1:**
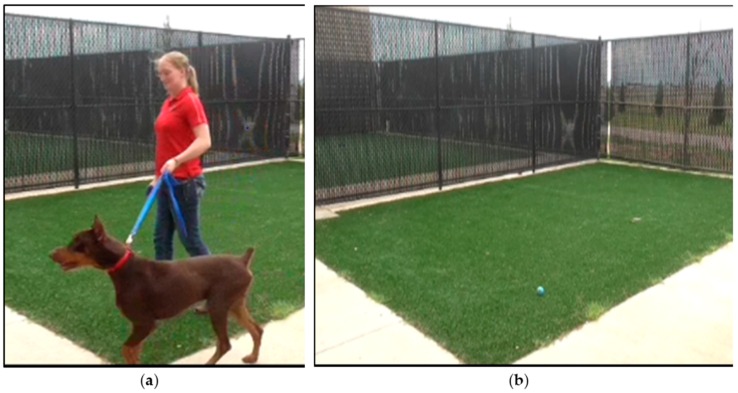
(**a**) Example of a dog being walked on a nylon leash and neck collar; (**b**) The yard in which dogs were walked.

Dogs were individually removed from their kennel and fit with a standard nylon neck collar with a plastic quick release closure and metal loop for leash attachment. A 2 m nylon leash was attached to the collar (Figure 1a). This type of leash and collar were chosen because they were a common type used by dog owners. A researcher walked each dog twice around a 7.9 m × 5.2 m fenced in yard covered with synthetic turf (total distance = 53 m; Figure 1b). On average, walks lasted approximately 76.0 ± 11.6 s. The yard was washed daily with a kennel disinfectant/deodorizer. 

The same female researcher walked every dog to eliminate variability in dog behavior caused by the human. During the walk, the leash was held so that the dog was kept within arm’s reach of the researcher. No verbal or visual cues were given by the researcher during the walk. A leash pull was defined as pressure exerted on the leash by the dog that resulted in the researcher’s inability to walk comfortably. The criteria chosen to define a leash pull was decided on based upon the fact that the sprays are marketed for use by the general public, and that body size and personal preference of each individual owner introduces a great amount of variability in what could be considered a leash pull. Walks were video-recorded (Panasonic HDC-TM90, Osaka, Japan), and each pull was signaled to the camera by the researcher raising her free arm. Dogs were permitted to pause for elimination; however, if a dog paused for longer than 15 s not for elimination, it was encouraged to move by a gentle tug on the leash. This tug was not counted as a leash pull. 

Treatments were applied on different days to avoid cross-contamination. Ten dogs were used for each treatment, and no dog received more than one treatment. Each dog was first walked with no correction given for leash pulling (NOTx). Dogs were permitted at least one hour of rest between the walk with no correction and the walk with the designated treatment. Treatments and associated abbreviations are defined in Table 2. Essentially, each NOT corresponds only to the associated treatment only (e.g., NOTA is used as the control for androstenone + water (ANH) only) in each comparison. Two consecutive walks with no treatment served as a negative control. Each time the dog pulled on the leash during the second walk, the researcher administered water only spray (H_2_O), androstenone diluted in a water-based spray (ANH), StopThat! (AND1), or InteroSTOP (AND2). ANH and AND2 each contained androstenone at a concentration of 1.0 µg/mL, and AND2 contained lavender fragrance (0.5% of inert ingredients) as well. AND1 contained androstenone at a concentration of 0.1 µg/mL and lavender fragrance (0.5% of inert ingredients). InteroSTOP and StopThat! sprays are both marketed by Perrigo Animal Health (Perrigo Animal Health, Omaha, NE, USA) as deterrent sprays, and are designed to elicit a disruptive stimulus in the form of a loud hiss of air. These sprays contain a pheromone that shows potential to modify dog behavior. ANH and H_2_O spray bottles had minimal noise and were adjusted to dispense comparable volumes of liquid with each spray. All treatments were sprayed 12 to 18 inches away from the dog’s head, which is the administration recommended by Perrigo Animal Health for InteroSTOP and StopThat! sprays. 

#### 2.2.2. Study 2: Jumping Up Model

Dogs remained in their home kennel while a researcher approached and stood in front of the kennel door. The researcher looked at the dog but gave no verbal or visual cues. Jumping up was defined as both front paws leaving the ground, ultimately resulting in the front paws resting on the kennel door, supporting the dog in an upright position (“up” position). This model is a variation of the barking model reported by McGlone *et al.* [17]. 

Ten dogs were used for each treatment, and no dog received more than one treatment. Treatments were applied on different days to prevent cross-contamination. Each dog experienced two trials at least 60 min apart. During the first trial, the researcher did not respond in any way to the dog jumping up (NOT). This served as the control for the treatment trial. In the treatment trials, each time the dog jumped the researcher sprayed the dog with water only (H_2_O), androstenone + water (ANH), androstenone 0.1 µg/mL, (AND1), or androstenone 1.0 µg/mL (AND2). Spray was administered 12 to 18 inches from the dog’s head. If the dog remained in the “up” position for 30 s, the researcher repeated the spray. Jumps were counted for 120 s following the first jump.

### 2.3. Data Collection

During Study 1, each trial was video-recorded and reviewed at Texas Tech University by trained observers blind to treatments. The number of leash pulls (as defined in Section 2.2.1) on each walk was counted. 

In Study 2, each trial was video-recorded and reviewed at Texas Tech University by trained observers blind to treatments. The number of jumps (as defined in Section 2.2.2) during the first and second 60 s period were counted and totaled, and the latency period in seconds between each jump was recorded. The number of dogs exhibiting avoidance of the researcher after the spray was counted. Avoidance is defined as intentionally moving away from the researcher and remaining at a distance of more than 1 m. 

### 2.4. Data Analyses

In the walking study, the primary measure was number of leash pulls. In the jumping up study, primary measures were number of jumps and latency between jumps, in seconds. Data were analyzed using the General Linear Models procedure of SAS (SAS Inst., Inc., Cary, NC, USA). Assumptions of GLM were met by this data, with equal variance between treatments and normal residual distribution in both models. Least Squares Means were separated by use of the predicted difference test within the General Linear Models program. Data from each study were analyzed to determine if sex had an effect; it did not and therefore sex was omitted from both models. Least Squares Means were calculated and compared for the number of leash pulls in the walking study, and for jumping up and the latency period in the jumping up study using treatment as the dependent variable. Differences among Least Squares Means were considered significant at *p* < 0.05. The NOT trial associated with each treatment group served as the control to which each treatment was compared. Independent predicted difference tests were conducted *post hoc* to compare Least Squares Means among treatments in each study, in particular to compared ANH, AND1, and AND2. A paired two-sample t-test was conducted to compare the number of jumps in the first and second minute of the jumping up model. A Pearson chi-square test was used to determine differences in the percent of dogs exhibiting avoidance behavior with each treatment. 

## 3. Results and Discussion

### 3.1. Study 1: Dog Walking Model

In this model, dogs did not significantly reduce pulling on the second walk due to fatigue or familiarity with the study area (NOT1 *vs.* NOT2, *p* = 0.39). When treated with H_2_O, dogs reduced pulling by 32.8% (*p* = 0.002); however, when treated with ANH leash pulling was reduced by 81.1% (*p* < 0.001). Because ANH combined the effects of spray plus the putative interomone, the effect of androstenone itself can be estimated by subtracting the effect of H_2_O from ANH. The data show that androstenone was responsible for reducing leash pulling by 48.3% more than the effect of just water. This demonstrates that androstenone reduced leash pulling behavior. AND1 and AND2 reduced leash pulling by 71.9% and 85.7%, respectively, compared to no correction (*p* < 0.001 and *p* = 0.002, respectively). Table 3 presents the number of leash pulls and the percent reduction for each treatment.

Independent predicted difference tests showed that ANH and AND2 each reduced leash pulling compared to H_2_O (*p* = 0.028 and *p* = 0.017, respectively). AND1 showed a tendency to reduce leash pulling compared to H_2_O (*p* = 0.054). Leash pulling was not different between ANH, AND1, and AND2. The lack of differences between ANH *vs.* AND1 or AND2 indicates that for leash pulling, the presence or absence of noise and different concentrations of androstenone did not change the behavior-altering effects of androstenone. The finding that androstenone reduces leash pulling is in agreement with the report that androstenone reduced barking [17].

### 3.2. Study 2: Jumping Up Model

All treatments were effective in reducing the number of jumps compared to NOT. Water reduced jumping by 79.3% compared to a >79.5% reduction in androstenone-based treatments. As with Study 1, the effect of androstenone alone was determined; in this study, androstenone accounted for only a 0.2% decrease in jumping up. Independent predicted difference tests showed no differences in the number of jumps among treatments (H2O, ANH, AND1, AND2). Results for that number of jumps are shown in Table 4. 

These results differ from the findings of Study 1 in that H_2_O was just as effective in reducing jumping up behavior as the androstenone-based treatments were. In this study, all sprays were more accurately directed toward the nose of the dog compared to Study 1; therefore, the finding that water alone was enough to deter jumping up suggests that a more direct spray may have been a stronger disruptive stimulus in this study. 

During the NOT trials, the dogs’ level of excitement appeared to wane after approximately 30 s, leading to fewer jumps in the second minute (2.4) than in the first minute (4.6) (*t* (49) = 2.01, *p* < 0.01). This is consistent with previous findings in a study conducted by Prato-Previde *et al.* [18], in which dogs were found to greet the owner (approach, tail-wagging, jumping up) more enthusiastically and for longer durations compared to a stranger [18]. Dogs associate the owner with positive attention such as treats, praise, and play; on the other hand, most dogs do not associate a strange person with either positive or negative attention and therefore greeting behaviors such as jumping up recede quickly. 

Data for the latency period is shown in Table 5. There was a treatment effect on the latency period between jumps for all treatments. Independent predicted difference tests showed that dogs treated with AND1 had the greatest increase in latency period (535.1%). ANH and AND2 increased the latency period by 350.3% and 352.3%, respectively. Independent *t*-tests showed that AND1 and AND2 were more effective than ANH in increasing the latency period (*p* = 0.016 and *p* < 0.001, respectively), suggesting that the burst of air is effective as a disruptive stimulus. These results are in disagreement with the findings of the walking study. We believe the reason for this disagreement between the studies can be explained by the angle of spray delivery. In the leash pulling study, the spray comes more from the side of the dog’s face, whereas in the jumping study all of the sprays were administered directly in front of the dog’s face. This more direct spray appears to provide enough of a deterrent in itself to effectively reduce jumping up.

After being sprayed with either H_2_O or ANH, 10% of dogs (*n* = 10 per treatment) exhibited avoidance behavior. On the other hand, 40% of dogs exhibited avoidance when sprayed with AND1 (χ^2^_(1, *n* = 10)_ = 24.0, *p* < 0.01) and 60% exhibited avoidance when sprayed with AND2 (χ^2^_(1, *n* = 10)_ = 54.95, *p* < 0.01). These observations, combined with evidence that the correction sprays were no more effective in reducing jumping up behavior, suggest that the burst of air intended as a disruptive stimulus may cause avoidance and perhaps fear. These corrections sprays should be used carefully or not at all when working with timid dogs or dogs prone to noise phobias.

## 4. Conclusions

The results of this study demonstrate that androstenone functions as an interomone based on the observation of behavioral changes elicited by exposure to the molecule. Dogs sprayed with androstenone reduced the number of leash pulls during a walk. In contrast, androstenone did not have a statistically significant effect on jumping up behavior beyond that elicited by a spray of water alone, although androstenone-treated dogs’ mean level of jumping up was lower than that of those treated with water. This suggests that the more direct application of sprays in the jumping up study compared to the leash pulling study is responsible for the disagreement of results. It seems that jumping up requires only a simple therapy (such as water) while leash pulling responds better to the interomone than to simply water. However, 40% and 60% of dogs sprayed with androstenone 0.1 µg/mL or androstenone 1.0 µg/mL, respectively, exhibited avoidance behavior. Based on evidence that water alone is effective in reducing jumping up, we caution the use of disruptive stimuli such as these correction sprays during training, especially in dogs that may have a timid disposition or that may be more sensitive to loud noises. In such dogs, the noise which is intended to be disruptive may actually be perceived as positive punishment, resulting in the pet exhibiting avoidance behavior toward the handler. For owners struggling to train dogs to walk properly on a leash, androstenone with or without the added noise of the spray may serve as an effective training tool in addition to its use as an immediate intervention to stop problem behaviors. This study was done in a controlled setting with few or no distractions, such as other animals or humans. Future work should be done with owners and pets to determine whether androstenone sprays are effective in a real-life situation. It is also important to bear in mind that positive reinforcement for desired behavior (walking loose-leash or sitting during greetings) is valuable and is a recommended aspect of any behavior training program. Although use of this interomone cannot be recommended for use against jumping up behavior, it may be useful in training dogs to walk properly on a leash.

## Figures and Tables

**Table 1:** Description of dogs used in the dog walking and leash pulling models.

Study	Age ^1^	Sex	Dogs (n)
Jumping Up (*n* = 40 dogs; 10 dogs per treatment)	Adult	Intact Female	6
	Adult	Spayed Female	7
	Adult	Intact Male	15
	Adult	Castrated Male	9
	Juvenile	Intact Female	1
	Juvenile	Spayed Female	1
	Juvenile	Castrated Male	1
Dog Walking (*n* = 50 dogs; 10 dogs per treatment)	Adult	Intact Female	8
	Adult	Spayed Female	10
	Adult	Intact Male	17
	Adult	Castrated Male	14
	Juvenile	Intact Female	1

**^1^** Adult indicates dog is >1 year of age. Juvenile indicates dog is <1 year of age.

**Table 2:** Walking study treatments and associated abbreviations. These abbreviations will be used for the remainder of this report.

First Walk	Abbreviation	Second Walk Treatment	Abbreviation	Androstenone Concentration	Aerosolized?
Nothing	NOT1	Nothing	NOT2	-	No
Nothing	NOTH	Water only	H_2_O	-	No
Nothing	NOTA	Androstenone + water	ANH	1.0 μg/mL	No
Nothing	NOTS	Androstenone 0.1 µg/mL	AND1	0.1 μg/mL	Yes
Nothing	NOTI	Androstenone 1.0 µg/mL	AND2	1.0 μg/mL	Yes

**Table 3:** Comparison of average number of leash pulls for dogs receiving no correction (NOT1) *vs.* no correction (NOT2), no correction (NOTH) *vs.* water only spray (H_2_O), no correction (NOTA) *vs.* androstenone + water spray (ANH), no correction (NOTS) *vs.* androstenone 0.1 µg/mL (AND1), or no correction (NOTI) *vs.* androstenone 1.0 µg/mL (AND2) in response to each pull. *n* = 10 dogs/treatment.

Treatment	Leash Pulls (#)	SEp of Leash Pulls	*p-*Value	Percent Reduction (%)
NOT1	8.7	0.72	0.57	12.6
NOT2	7.6 **^a^**			
NOTH	6.4	0.57	0.11	32.8
H_2_O	4.3 **^b^**			
NOTA	7.4	0.40	<0.01	81.1
ANH	1.4 **^c^**			
NOTS	6.4	0.73	<0.01	71.9
AND1	1.8 ^**b**,**c**^			
NOTI	8.4	0.37	<0.01	85.7
AND2	1.2 **^c^**			

^**a**,**b**,**c**^ Least Squares means of treatments with differing superscripts are different, *p* < 0.05. **#** = average number of leash pulls per walk.

**Table 4:** Comparison of average number of jumps for dogs receiving no correction (NOTH) *vs.* water-only spray (H_2_O), no correction (NOTA) *vs.* androstenone + water spray (ANH), no correction (NOTS) *vs.* androstenone 0.1 µg/mL (AND1), or no correction (NOTI) *vs.* androstenone 1.0 µg/mL (AND2) in response to each instance of jumping up. *n* = 10 dogs/treatment.

Treatment	Jumps (#)	SEp	*p*-Value	Percent Reduction (%)
NOTH	11.1	1.94	<0.01	79.3
H_2_O	2.3			
NOTA	7.8	1.94	0.03	79.5
ANH	1.6			
NOTS	8.6	1.94	0.01	84.9
AND1	1.3			
NOTI	6.1	1.94	0.07	82.0
AND2	1.1			

Notes: Each treatment has an associated NOT value. **#** = average number of leash pulls per walk.

**Table 5:** Comparison of latency period between jumps for dogs receiving no correction (NOTH) *vs.* water-only spray (H_2_O), no correction (NOTA) *vs.* androstenone + water spray (ANH), androstenone 0.1 µg/mL (AND1), or no correction (NOTI) *vs.* androstenone 1.0 µg/mL (AND2) in response to each instance of jumping up. *n* = 10 dogs/treatment.

Treatment	Latency Period (s)	Standard Error	*p*-Value	Percent Increase (%)
NOTH	15.7	8.31	<0.01	340.8
H_2_O	69.2 **^a^**			
NOTA	17.7	8.31	<0.01	350.3
ANH	79.7 **^a^**			
NOTS	17.1	8.31	<0.01	535.1
AND1	108.6 **^b^**			
NOTI	26.2	8.31	<0.01	352.3
AND2	118.5 **^b^**			

Notes: each treatment has an associated NOT value. **^a^**^,**b**^ Least Squares means of treatments with differing superscripts are different, *p* < 0.05.
